# SARS-CoV-2 in diabetic pregnancies: a systematic scoping review

**DOI:** 10.1186/s12884-021-03975-3

**Published:** 2021-08-21

**Authors:** Claudia Eberle, Tamarra James-Todd, Stefanie Stichling

**Affiliations:** 1 Medicine with specialization in Internal Medicine and General Medicine, Hochschule Fulda - University of Applied Sciences, Fulda, Germany; 2 Department of Environmental Health, Harvard T.H. Chan School of Public Health, Boston, MA USA; 3 Department of Epidemiology, Harvard T.H. Chan School of Public Health, Boston, MA USA

**Keywords:** Diabetes, Pregnancy, COVID-19, SARS-COV-2, Systematic review

## Abstract

**Background:**

Currently, we suffer from an increasing diabetes pandemic and on the other hand from the SARS-CoV-2 pandemic. Already at the beginning of the SARS-CoV-2 pandemic, it was quickly assumed that certain groups are at increased risk to suffer from a severe course of COVID-19. There are serious concerns regarding potential adverse effects on maternal, fetal, and neonatal outcomes. Diabetic pregnancies clearly need special care, but clinical implications as well as the complex interplay of diabetes and SARS-CoV-2 are currently unknown. We summarized the evidence on SARS-CoV-2 in diabetic pregnancies, including the identification of novel potential pathophysiological mechanisms and interactions as well as clinical outcomes and features, screening, and management approaches.

**Methods:**

We carried out a systematic scoping review in MEDLINE (PubMed), EMBASE, CINAHL, Cochrane Library, and Web of Science Core Collection in September 2020.

**Results:**

We found that the prognosis of pregnant women with diabetes mellitus and COVID-19 may be associated with potential underlying mechanisms such as a simplified viral uptake by ACE2, a higher basal value of pro-inflammatory cytokines, being hypoxemic as well as platelet activation, embolism, and preeclampsia. In the context of “trans-generational programming” and COVID-19, life-long consequences may be “programmed” during gestation by pro-inflammation, hypoxia, over- or under-expression of transporters and enzymes, and epigenetic modifications based on changes in the intra-uterine milieu. COVID-19 may cause new onset diabetes mellitus, and that vertical transmission from mother to baby might be possible.

**Conclusions:**

Given the challenges in clinical management, the complex interplay between COVID-19 and diabetic pregnancies, evidence-based recommendations are urgently needed. Digital medicine is a future-oriented and effective approach in the context of clinical diabetes management. We anticipate our review to be a starting point to understand and analyze mechanisms and epidemiology to most effectively treat women with SARS-COV-2 and diabetes in pregnancy.

**Supplementary Information:**

The online version contains supplementary material available at 10.1186/s12884-021-03975-3.

## Background

Currently, we face two pandemics at the same time. On the one hand, we suffer from an increasing diabetes mellitus (DM) pandemic and on the other hand from the severe acute respiratory syndrome coronavirus 2 (SARS-CoV-2) pandemic. By focusing on the WHO, 67,530,912 million people are diagnosed with COVID-19, globally, including 1,545,140 deaths deaths (as of December 09, 2020) [1]. Already at the beginning of the SARS-CoV-2 pandemic, it was quickly assumed that certain groups such as elderly, pregnant women and those with multiple or chronic comorbidities, such as diabetes mellitus, are more likely to get infected and are at increased risk to suffer from a severe course of Coronavirus Disease 2019 (COVID-19)[2–4]. Hence, pregnant women become a particularly vulnerable group if they are diagnosed with diabetes mellitus and are also infected with the novel corona virus. Very up to date, there are serious concerns regarding potential adverse effects on maternal, fetal, and neonatal outcomes [5] and that SARS-CoV-2 might be vertically transmitted [6]. Therefore, diabetic pregnancies clearly need special care, but, clinical implications of COVID-19 are currently unknown [7].

Referring to the International Diabetes Federation (IDF), 463 million people are diagnosed with diabetes mellitus in 2019, worldwide [8]. National and international scientific committees call diabetes mellitus a metabolic pandemic [9–11], which is clearly associated with increased comorbidities and complications, such as obesity, Metabolic Syndrome (MetS), cardiovascular disease (CD), increased morbidity, hospitalizations, mortality as well as emerging costs for the global health system, in general [9]. In the context of the diabetes pandemic, pregnancies complicated by diabetes are occurring worldwide, with the result that hyperglycemia in pregnancy (HIP) has the fastest growing prevalence. In 2019 and with reference to the IDF, approximately 20 million (16%) of live births suffered from some form of HIP [8]. Looking closer, approx. 84% of HIP were defined as gestational diabetes mellitus (GDM), approx. 7.9% were diagnosed prior to pregnancy with type 1 diabetes (T1D) and type 2 diabetes (T2D), and approximately 8.5% were first observed with T1D and T2D in pregnancy [8]. Depending on the country, the prevalence of GDM ranged between approximately 2.1% and approximately 37.5% in 2019 [8]. In Europe, the prevalence of GDM is estimated at about 16.3%, whereas the prevalence in North America and Caribbean is approximately 20.8%, and 27.0% in South East Asia [8]. In all cases, the estimated number of unreported cases is not included. Moreover, GDM is mostly diagnosed in the second or third trimester and defined as not clearly overt diabetes prior to gestation [12]. Although there are various regional variations and different screening and diagnosis guidelines worldwide, risk factors leading to GDM, such as maternal age, obesity, rising gestational weight gain, seem to be the same, globally [13, 14]. Gestational diabetes mellitus is clearly associated with adverse pregnancy and birth (short-term) outcomes for mother and offspring, e.g. increased risks of preeclampsia, hypoxia, caesarean sections, macrosomia and shoulder dystocia, hypoglycemia [13, 15]. Furthermore, GDM is closely associated with adverse long-term outcomes in mothers and children, such as a subsequent increased risk of T2D, obesity, MetS and CD or even depression [15–17].

**Fig. 1:**
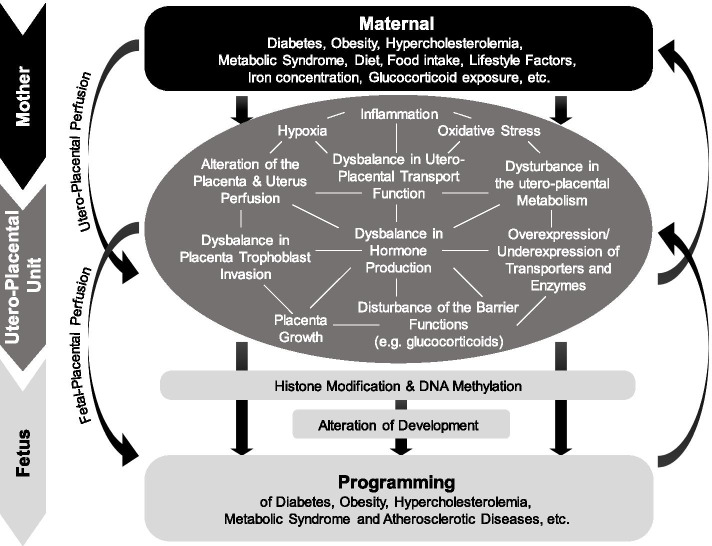
Mechanisms of fetal programming (Eberle and Ament 2012[1]). References: 1. Eberle C, Ament C. Diabetic and metabolic programming: mechanisms altering the intrauterine milieu. ISRN pediatrics. 2012; 2012:975,685. https://doi.org/10.5402/2012/975685 PMID: 23,213,562

Having the concepts and mechanisms of trans-generational programming (“fetal, perinatal and in utero programming”) in mind, intra-uterine exposure to increased levels of hyperglycemia “program” the offspring for life-long consequences to e.g. obesity, glucose intolerance, T2D, insulin resistance, MetS, high blood pressure, CD early on [18–24]. In a side note, trans-generational programming (or “fetal programming”, “perinatal programming”), means a perturbation during prenatal development phase, which may lead to dysfunctions in organs and metabolic regulation, that, in turn, might lead to life-long diseases like impaired glucose intolerance (IGT), DM, obesity, MetS, CD, etc. in later life [21, 22, 24–26]. These life-long consequences may be “programmed” during (pre-)gestation by different molecular mechanisms, such as pro-inflammation, hypoxia, oxidative stress, over- or underexpression of transporters and enzymes, dysbalance in hormone production as well as epigenetic modifications, such as Histone modification and DNA methylation, based on changes in the intra-uterine milieu (Fig. 1) [22].

Analyzes of the transgenerational and complex interplay of COVID-19, diabetes mellitus and pregnancy are urgently needed. The purpose of this systematic scoping review was to summarize the current literature on SARS-CoV-2 in diabetic pregnancies, including identification of novel potential pathophysiological mechanisms and interactions as well as clinical outcomes and features, screening, and management approaches.

## Methods

### Data sources and search strategy

We carried out a systematic scoping review to outline potential pathophysiological mechanisms, examine clinical outcomes and review different types of evidence. A systematic scoping review follows the steps of a systematic review but with a broader scope [27]. We followed PRISMA for systematic reviews [28] and Joanna Briggs Institute for systematic scoping reviews guidelines [27].

We carried out literature searches on: COVID-19 and Diabetes Mellitus (I), COVID-19 and pregnancy (II), and COVID-19 and diabetic pregnancies (III). We searched MEDLINE (PubMed), EMBASE, CINAHL, Cochrane Library, and Web of Science Core Collection databases for studies and arcticles published until September 2020. In addition, reference lists and Google Scholar were searched manually. Our search strategy is provided in Supplementary Table 1. No study protocol has been published. After removing duplicates, titles and abstracts were screening according to our eligibility criteria. Suitable articles were selected by two independent reviewers.

### Eligibility

We included different study designs (reviews, meta-analyzes, clinical trials, epidemiological studies) and article types (comments, letters, notes, case reports) on the topics of COVID-19 and pregnancy/ diabetes mellitus with regard to pathophysiological mechanisms, clinical outcomes and features, screening, and management. We involved articles in English and German published until September 2020.

### Data extraction

In addition to authors, location and year, we extracted key results and information on the subtopics (pathophysiological mechanisms, clinical outcomes and features, screening, and management), and if available sample sizes, outcomes, and types of diabetes. Conforming to the guidelines for (systematic) scoping reviews, we aimed at providing an overview of the evidence and reviewing different types of evidence regardless of quality, hence no formal quality appraisal was was conducted.

## Results

We identified *n* = 1,938 citations without duplicates for COVID-19 and Diabetes Mellitus, *n* = 985 for COVID-19 and pregnancy, *n* = 70 for COVID-19 and GDM/diabetic pregnancies. In total, we screened *n* = 3,003 citations based on titles/abstracts (*n* = 10 were added through manual research), and selected *n* = 49 articles based on our eligibility criteria. The PRISMA flow chart is shown in Supplementary Figure 1. Supplementary Table 2 provides a list of the included studies.

### SARS-CoV-2 and diabetes mellitus

While COVID-19 passes asymptomatically in some cases, the course of the disease in others can be very severe [2]. There are patients diagnosed by a positive RT–PCR test but are asymptomatic or only minimally symptomatic [29]. A meta-analysis by Yang et al. indicated that DM (8 ± 6%, 95% CI 6% to 11%) and CD (5 ± 4%, 95% CI 4%-7%) were the most common comorbidities observed in SARS-CoV-2 patients [30]. According to another meta-analysis by Li et al., the incidence of DM was about twice as high in severe cases of COVID-19 (intensive care unit) than in non-severe counterparts [31]. DM was also clearly associated with intensive care unit admission for COVID-19 (OR: 2.06; 95%CI 1.09–3.92, *P* = 0.027) and a longer length of hospital stay in a cohort study by Al-Salameh et al. [32]. Factors that moderate the relationship between SARS-CoV-2 and DM are currently unclear, but, an increased BMI, as well as HbA1c level, might be linked with worse outcomes in patients diagnosed with DM and COVID-19 [33]. It is not yet clear what exactly causes the increased risk of patients diagnosed with DM. There are different approaches to this.

The predisposition of DM to a severe course could be explained by comorbidities of the MetS such as arterial hypertension, dyslipoproteinemia, visceral obesity and non-alcoholic hepatic steatosis. In a retrospective analysis by Guo et al. of *n* = 174 patients with COVID-19, patients with DM (without further comorbidities) had a higher risk for severe pneumonia, release of tissue injury-related enzymes, excessive uncontrolled inflammation responses and hypercoagulable state associated with dysregulation of glucose metabolism [34]. In addition, serum levels of inflammation-related biomarkers such as IL-6, C-reactive protein, serum ferritin and coagulation index, D-dimer, were clearly higher in patients diagnosed with DM [34]. Moreover, an altered immune response in patients diagnosed with DM, possibly due to impaired lymphocyte, neutrophil and monocyte/macrophage function, might be an underlying mechanism of higher risk of severe COVID-19 progression [35]. In a retrospective study by Chen et al. involving *n* = 904 patients, 15% of the enrolled COVID-19 patients were diagnosed with DM and also higher levels of D-dimer, while female patients had increased lactate dehydrogenase (LDH) and neutrophil counts [36]. A risk factor for higher mortality of patients diagnosed with DM and COVID-19 was, among others, elevated C-reactive protein (aOR 1.12 [95% CI 1.00, 1.24]; *P* = 0.043), implicating C-reactive protein may identify high risk patients during hospitalization [36].

In general, DM increases the susceptibility to infections. Insulin resistance might reduce T cell activity and thus lead to a weaker immune response [37]. The innate immune response could be affected by the formation of advanced glycation end products. Elevated insulin levels are also associated with an increased risk of prothrombotic events [38]. The increased risk of thrombosis and the pathological changes to the vascular endothelium are factors that are associated with a severe course of COVID-19 [39].

Overall, there are clear hints that the prognosis of patients diagnosed with COVID-19 and DM may be associated with a simplified viral uptake by receptor angiotensin-converting enzyme 2 (ACE2), with a higher basal value of pro-inflammatory cytokines which facilitate a cytokine storm, with being hypoxemic and with elevated levels of IL-6 and AMPK/mTOR signaling pathway [40, 41].

### SARS-CoV-2 and pregnancy

According to a meta-analysis by Allotey et al., the rate of COVID-19 diagnoses in pregnant women attending or admitted to hospitals is approx. 10% (95% confidence interval (CI): 7% to 14%) (*n* = 11,432 women from *n* = 26 studies) [42]. Pre-existing diabetes [OR 2.12 (95% CI: 1.62 to 2.78)] was associated with severe COVID-19 in pregnancy, while GDM was not [OR 2.12 (95% CI: 1.61 to 2.78)] [42].

Currently, limited data on maternal, fetal and neonatal outcomes of pregnant women with SARS-CoV-2 infection are available; however, the data that exists suggest higher risks of pregnancy complications including preterm birth and preeclampsia [42, 43]. Pregnant women probably need more intensive care and treatment for COVID-19 [42, 44]. Risk factors for severe COVID-19 in pregnant women include comorbid conditions, high body mass index (BMI), and high maternal age [42].

A systematic review by Zaigham and Andersson [45] analyzing *n* = 108 pregnancies reported that women (third trimester) presented fever (68%), coughing (34%), and lymphocytopenia (59%) with elevated C‐reactive protein (70%) as well as 91% of the women were delivered by cesarean section. The authors suggest that fetal distress was reported to as the indication for cesarean section. In addition, one neonatal and one intrauterine death were observed [45]. A small analysis by Schwartz (Zhongnan Hospital, Wuhan) indicated elevated C-reactive protein (6 of 9 women), lymphopenia (5 of 9), increased alanine aminotransferase and aspartate aminotransferase (3 of 9), preterm labor (4 of 9), fetal distress (5 of 8), premature rupture of membranes (7 of 9), cesarean deliveries (9 of 9). In addition, no cases of severe pneumonia and maternal death were observed and all infants had good Apgar Scores. Tests for the presence of SARS-CoV-2 were negative regarding amniotic fluid, breastmilk, umbilical cord blood, and neonatal throat swabs [46]. In another small study by Liu et al. (*n* = 13 women) reported that 46% had preterm labour (32–36 weeks of gestation) and 77% underwent caesarean section, with half being emergency cesarean section due to pregnancy complications including fetal distress (3 of 10), premature rupture of the membrane (1 of 10) and stillbirth (1 of 10) [47].

### Potential mechanisms

#### Angiotensin-Converting Enzyme 2 (ACE2)

The Spike glycoprotein on the surface of SARS-CoV-2 binds to ACE2 receptors, which are expressed in main metabolic organs and tissues, for example, pancreatic beta cells, adipose tissue, the small intestine, and kidneys [48]. Therefore, SARS-CoV-2 may cause alterations of glucose metabolism complicating the pathophysiology of preexisting DM or leading to new mechanisms [48]. An overexpression of ACE2 seems to be counterproductive in COVID-19 [49] [50]. However, the current evidence is insufficient and suggests no modifications of the treatment [50]. Caution, for fertility phase and pregnancy there are different approaches. First evidence showed that SARS-CoV-2 might infect the placenta and thus influence the intrauterine environment. Hecht et al. reported that ACE2 is expressed in the trophoblast of the placenta with a polarized membranous pattern in the syncytiotrophoblast, and that transmembrane protease serine subtype 2 (TMPRSS2) is expressed weakly mainly in the chorionic villous endothelium. The authors demonstrated SARS-CoV-2 virus infection of the syncytiotrophoblast and cytotrophoblast and the possibility of vertical transmission [51]. Other reports also support the findings regarding COVID-19, placental infection and vertical transmission [52–54].

#### Pro-Inflammation

Pregnancy is a pro-inflammatory state. Even regular third-trimester pregnancies stand out by increased levels of peripheral blood leukocytes, which are boosted by preeclampsia[55]. According to Wei et al., levels of pro‐inflammatory cytokines are markedly increased in COVID‐19 patients and linked to disease progression [56]. Referring to Pal and Bhadada, DM is also a pro-inflammatory state, and COVID-19 patients with DM showed significantly higher serum levels of interleukin-6 (IL-6), C-reactive protein and ferritin than those without DM [57]. Therefore, the authors conclude that patients who are diagnosed with DM are more susceptible to inflammatory cytokine storms leading to deterioration of COVID-19. Furthermore, a study from Wuhan indicated that 10% of patients with T2D and COVID-19 had at least one hypoglycemia episode [58], leading to cardiovascular events by mobilizing pro-inflammatory mononuclear cells and increasing platelet reactivity, amongst other things [57]. Therefore, COVID-19 leads to deteriorating the glycemic profile that supports generation of pro-inflammatory cytokines leading to a vicious cycle [57].

Immunological states in pregnancy actively modify with the growth of the fetus from pro-inflammatory state in the first, to anti-inflammatory in the second, and to second pro-inflammatory state in the third trimester [59]. Having this in mind, the hypothesized cytokine-storm caused by SARS-CoV-2 may lead to more severe inflammatory state in pregnant women, resulting in severe morbidity and mortality. In addition, maternal inflammation as a result of viral infection can affect fetal brain development and neuronal functions [59].

#### Hypoxia and preeclampsia

Hypoxia is a major clinical outcome in COVID-19 patients and current evidence suggests that COVID-19 has an impact on the cardiovascular system by hypoxia, among other things [60]. Pulmonary inflammation and dysfunction caused by SARS‐CoV‐2 infection triggers hypoxaemia [56]. A study by Xie et al. identified *n* = 20 (of *n* = 140; 14.29%) COVID-19 patients with DM [41]. Of these, *n* = 8 had hypoxemia (SpO2 ≤ 90%) (and *n* = 51 COVID-19 patients in total) and hypoxemia was independently associated with in-hospital mortality [41]*.*

Hyper-inflammatory state in COVID-19 may be associated with hypoxic injury in the placenta and developing pre-eclamptic state. A potential SARS-CoV-2 intra-uterine infection may alter the expression of ACE2 and generate pre-eclamptic state by increased Angiotensin II level in the placental villi leading to vasoconstriction and restricted fetal blood flow [61].

When binding to ACE2, SARS causes its downregulation and lowering angiotensin-(1–7) levels. This can worsen vasoconstriction, inflammation, and pro-coagulopathic effects that occur in preeclampsia. Limited evidence indicates that preeclampsia may be more common in pregnant COVID-19 patients [43].

### SARS-CoV-2 and new-onset (Gestational) diabetes mellitus?

Metabolic imbalances can occur with severe viral diseases. For instance, Hepatitis C infection is a risk factor for T2D, associated with beta cell dysfunction [62]. Metabolic complications specific to COVID-19 are not yet well characterized [62]. SARS coronavirus enters islets using ACE2 as its receptor and can damage islets causing DM [63]. There is emerging evidence that COVID-19 may cause new-onset DM and severe metabolic complications of pre-existing DM via ACE2 (insulin deficiency and increased risk of Diabetic Keto-Acidosis) [48, 62], which leads to challenges in clinical management and requires further investigation. Whether COVID-19 can trigger a new DM requires further research. Based on these initial assumptions, it is conceivable that COVID-19 could also trigger GDM, but currently there are no studies in this regard.

### SARS-CoV-2 – perinatal programming in diabetic pregnancies?

Following the concepts of “perinatal programming”, SARS-COV-2 and its complications may affect and change intrauterine milieu. Even a moderate degree of hypoxemia may affect the fetal development and organism profoundly [64]. A recent study examined a placenta showing signs of acute and chronic inflammation consistent with the severe maternal inflammatory status initiated by SARS-CoV-2 infection. Transmission through placenta was clearly observed [65]. First reports suggest vertical transmission from mother to baby, which needs further research [6]. However, it should be taken into account that vertical transmission of the virus is controversial, with evidences considering it a rare event, and postnatal infection of the newborn from a mother infected peripartum is more likely [66].

**Fig. 2:**
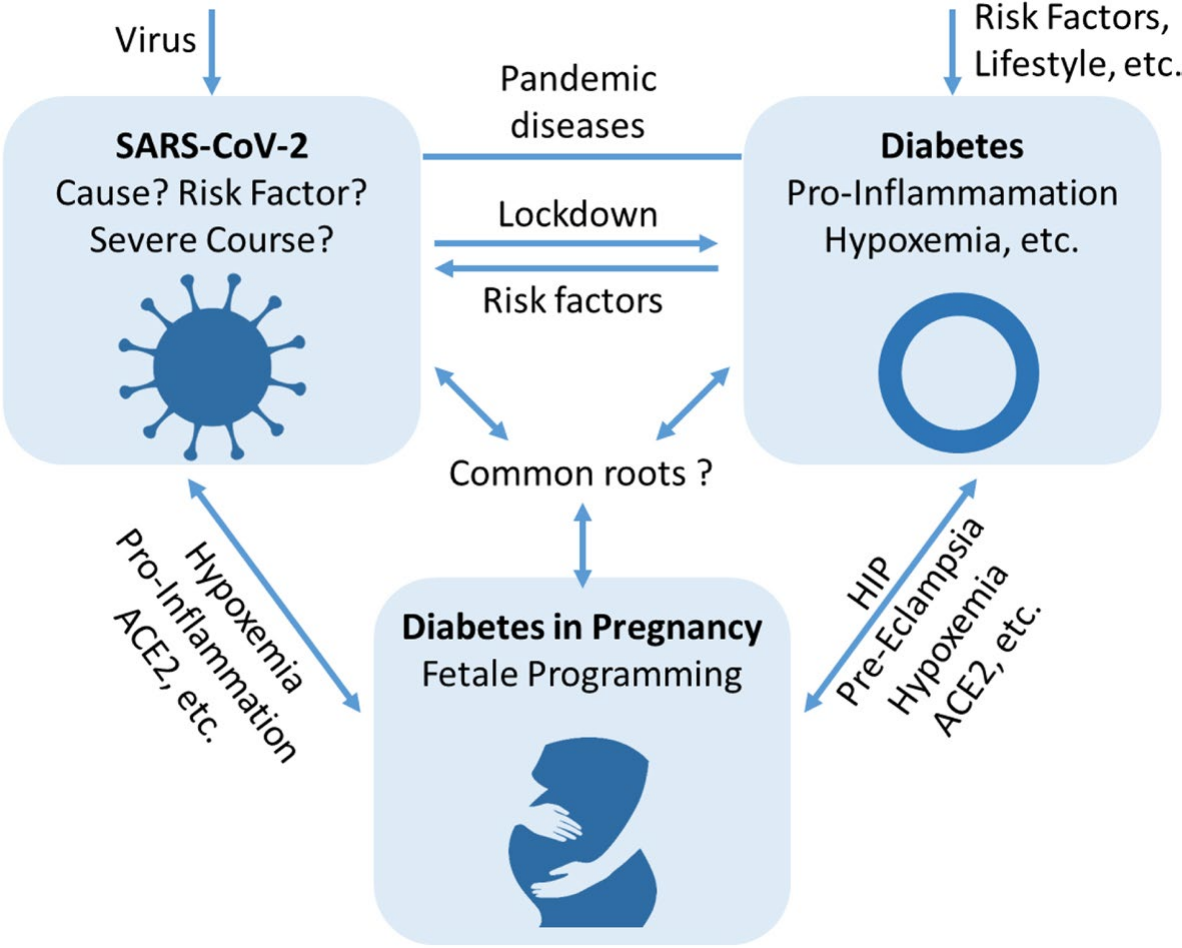
Interplay of SARS-CoV-2, diabetes mellitus, and pregnancy

Based on mechanisms of diabetic programming, maternal hyperglycemia causes similar intra-uterine changes by hypoxemia and pro-inflammation leading to life-long consequences in the offspring (Fig. 1) [22]. Therefore, SARS-CoV-2 may cause maternal viremia, placental infection, placental inflammation and neonatal viremia (Fig. 2).

### SARS-CoV-2 and screening/management of (Gestational) diabetes mellitus

Not only DM but also pregnancy per se predisposes women to viral infection [5]. Pregnant COVID-19 patients with DM are a particularly vulnerable group. Adverse effects on maternal, fetal, and neonatal outcomes seem possible [5]. Decontamination recommendations for pregnant women suggest mask wearing, good hand hygiene, and social distancing in order to best protect pregnant women from infection [73]. Furthermore, there are ethnic and socioeconomic differences in risk of COVID-19 hospitalisation and infection [74, 75].

Due to the COVID-19 pandemic and infection control measures, people with DM may be concerned by limited healthcare, medication, healthy diet, and healthy lifestyle access [67]. In the context of COVID-19 and GDM, pregnant women and health care professionals are rather unwilling to perform the Oral Glucose Tolerance Test (OGTT) because of travelling, the need for two visits, and exposure to potentially infectious environment. Therefore, professional societies (e.g. from United Kingdom, Canada, Australia, New Zealand) modified GDM screening during COVID-19 pandemic and recommended telemetric support by health care professionals to control fasting blood glucose (FBG), glycated hemoglobin (HbA1c), and random plasma glucose (RPG) as alternative tests at 24–28 weeks of gestation [68, 69]. In addition, the International Association of Diabetes and Pregnancy Study Groups (IADPSG) recommend the “one step” approach with 2 h, 75 g OGTT after overnight fasting of 8–14 h [70]. According to the IADPSG criteria, the 75 g OGTT can be dispensed during the pandemic if the fasting glucose level is elevated [70]. These novel alternative recommendations, however, are not yet evidence-based and may also result in only women with a high risk being identified and treated [69]. Van‐de‐l’Isle et al. (cohort study, UK) [71] reported that the Royal College of Obstetricians and Gynaecologists (RCOG) COVID‐19 GDM screening regime (FBG ≥ 5.3 mmol/L or HbA1c ≥ 5.7%) did not detect 57% (47 of 82) women subsequently identified as gestational diabetics. Moreover, Zhu et al. [72] (retrospective study, Australia) indicated that 25.3% (60 of 237) patients would not have had GDM detected using the Australasian Diabetes in Pregnancy Society (ADIPS) guidelines (FBG ≥ 5.1 mmol/L and/or HbA1c ≥ 5.9%).

### SARS-CoV-2 and digital medicine

In the context of the COVID-19 pandemic, the sudden shift to digital approaches, such as telemedicine, was taking place in medical practices globally and brought to the forefront of medical care [67, 73–75]. From a digital perspective, there are several novel therapy approaches, but telemedicine has become the most effective in the current situation. “Digital care”, including telemetry, represents an innovative, promising, and effective approach to optimize prenatal care in the setting of the COVID-19 pandemic [76, 77]. Telemetric interventions have shown clearly improved care in diabetic pregnancies [78]. “Digital care” is effective in monitoring patients after discharge, visiting susceptible patients such as pregnant women diagnosed with DM, protecting health care professionals, and collecting data from the isolation period during the pandemic [79]. Within the context of closed-meshed medical support, “digital care” works for patients and health care professionals just as well in the shape of mobile devices, such as smartphone-Apps [80] – especially for pregnant women [81–83]. We found one study [84] examing the GDM management using a smartphone application with artificial intelligence during the COVID-19 pandemic. This app automatically analyzes the patient data and made recommendations, monitored and adjusted by health care professionals. The author concluded that the app is an innovative tool to prevent unnecessary hospital visits while keeping the best quality healthcare [84].

## Conclusions

Although various COVID-19 registers have already been set up (CRONOS – COVID-19 Related Obstetric and Neonatal Outcome Study, Germany; COVID-19 PRIORITY – Pregnancy CoRonavIrus Outcomes RegIsTrY, USA; COVID-NET – A Weekly Summary of U.S. COVID-19 Hospitalization Data, USA; New Zealand Registry of Covid-19 in Pregnancy; CHOPAN –Coronavirus Health Outcomes in Pregnancy and Newborns, Australia; PregCOV-19LSR – COVID-19 in Pregnancy Living Systematic Reviews, UK), the published data are currently insufficient. In general, pregnant women with diabetes mellitus are more likely to get infected and are at an increased risk of a severe course of COVID-19. The prognosis of pregnant women with DM and COVID-19 may be associated with potential underlying mechanisms such as a simplified viral uptake by ACE2, a higher basal value of pro-inflammatory cytokines, being hypoxemic as well as platelet activation, embolism, and preeclampsia. In the context of “trans-generational programming” and COVID-19, life-long consequences may be “programmed” during gestation by pro-inflammation, hypoxia, over- or under-expression of transporters and enzymes, and epigenetic modifications based on changes in the intra-uterine milieu. Furthermore, first evidence suggests that COVID-19 may cause new onset diabetes mellitus, and that vertical transmission from mother to baby might be possible. Given the challenges in clinical management, the complex and trans-generational interplay between COVID-19 and diabetic pregnancies, further research and evidence-based recommendations are urgently needed. Moreover, digital medicine is a future-oriented and effective approach in the context of clinical diabetes management, particularly in the face of the current syndemic, the possibilities of which we need to better understand and analyze.

One limitation is the language restriction. Since this is a special and rapidly developing topic, we have included different article types such as comments, letters, etc. and generally many different study designs. Since there is only a small amount of literature on the topic so far, we have decided on a systematic scoping review, which makes it possible to show a broad range of evidence regardless of quality and type and serves as an overview and starting point for further research in the individual subtopics. Further research is urgently needed to understand the mechanisms, epidemiology, and health care systems and infrastructures needed to most effectively prevent, diagnose and treat women with SARS-COV-2 and diabetes in pregnancy.

## Supplementary Information

**Additional file 1.**

## Data Availability

All data generated or analysed during this study are included in this published article and its supplementary information files.

## Abbreviations

GDM: Gestational diabetes mellitus
DM: Diabetes mellitus
SARS-CoV-2: Severe acute respiratory syndrome coronavirus 2
COVID-19: Coronavirus Disease 2019
T1DM: Type 1 diabetes mellitus
T2DM: Type 2 diabetes mellitus
MetS: Metabolic Syndrome
CD: Cardiovascular disease
ACE2: Angiotensin-Converting Enzyme 2
