# Supplementary material for: SARS-CoV-2 in diabetic pregnancies: a systematic scoping review
Source: BMC Pregnancy Childbirth. 2021 Aug 21;21:573. doi: 10.1186/s12884-021-03975-3 (PMC8379032; doi:10.1186/s12884-021-03975-3)
Supplement: Supplementary file 1 — Additional file 1. [file 12884_2021_3975_MOESM1_ESM.docx]

**SUPPLEMENTARY INFORMATION**

SARS-CoV-2 in Diabetic Pregnancies: A SYSTEMATIC SCOPING REVIEW

Claudia Eberle, M.D.^1^, Tamarra James-Todd^2^, Stefanie Stichling, M.Sc.^1^

**Table of Contents**

Search strategies page 2

Included studies pages 3-6

PRISMA flow chart page 7

# *Supplementary Table 1. Search strategies.*

|  | PUBMED | EMBASE | COCHRANE LIBRARY | CINAHL | WEB OF SCIENCE  CORE COLLECTION | GOOGLE SCHOLAR |
| --- | --- | --- | --- | --- | --- | --- |
| KEYWORDS | COVID-19, SARS-CoV-2, diabetes mellitus, pregnancy, gestational diabetes mellitus | coronavirus disease 2019, severe acute respiratory syndrome coronavirus, pregnancy diabetes mellitus | COVID-19, SARS-CoV-2, coronavirus, diabetes mellitus, pregnancy, gestational diabetes mellitus | COVID-19, SARS-CoV-2, diabetes mellitus, pregnancy | COVID-19, SARS-CoV-2, diabetes mellitus, pregnancy, gestational diabetes mellitus | COVID-19, SARS-CoV-2, diabetes mellitus, pregnancy, gestational diabetes mellitus |
| FILTERS | Published until 2020/09/10,  German and English | Published until 2020/09/10  German and English, [embase]/lim | Published until 2020/09/10,  German and English | Published until 2020/09/10,  German and English | Published until 2020/09/10,  German and English | Published until 2020/09/10,  German and English |
| STRATEGIES | (("SARS-COV-2"[Title/Abstract]) OR ("COVID-19" [Supplementary Concept])) AND (diabetes mellitus[MeSH Terms]) **n=396** | ((‘diabetes mellitus’/exp) AND (‘coronavirus disease 2019’/exp OR 'severe acute respiratory syndrome coronavirus 2'/exp))  **n=1720** | ((MeSH [Diabetes Mellitus]) AND (“COVID-19”:ti,ab OR “SARS-CoV-2”:ti,ab OR “coronavirus”:ti,ab)  **n=8** | ((MH “diabetes mellitus”) AND (TI “SARS-CoV-2” OR AB “SARS-CoV-2” OR MH “COVID-19))  **n=63** | ((TOPIC “diabetes mellitus”) AND (TOPIC “COVID-19” OR TOPIC “SARS-CoV-2”))  **n=195** | Additional manual research using keywords in different combinations  **n=6** |
|  | (("SARS-COV-2"[Title/Abstract]) OR ("COVID-19" [Supplementary Concept])) AND (pregnancy[MeSH Terms])  **n=523** | ((‘pregnancy’/exp) AND (‘coronavirus disease 2019’/exp OR 'severe acute respiratory syndrome coronavirus 2'/exp))  **n=463** | ((MeSH [pregnancy]) AND (“COVID-19”:ti,ab OR “SARS-CoV-2”:ti,ab OR “coronavirus”:ti,ab))  **n=0** | ((MH “pregnancy”) AND (TI “SARS-CoV-2” OR AB “SARS-CoV-2” OR MH “COVID-19)) **n=149** | ((TOPIC “pregnancy”) AND (TOPIC “COVID-19” OR TOPIC “SARS-CoV-2”)) **n=381** |  |
|  | (((pregnancy[MeSH Terms]) AND (diabetes mellitus[MeSH Terms])) OR (gestational diabetes mellitus[MeSH Terms])) AND (("SARS-COV-2"[Title/Abstract]) OR ("COVID-19" [Supplementary Concept])) **n=10** | ((‘pregnancy diabetes mellitus’) AND (‘coronavirus disease 2019’/exp OR 'severe acute respiratory syndrome coronavirus 2'/exp))  **n=49** | ((MeSH [Diabetes, Gestational]) AND (“COVID-19”:ti,ab OR “SARS-CoV-2”:ti,ab OR “coronavirus”:ti,ab)) **n=0** | ((MH “pregnancy” AND (MH “diabetes mellitus”) AND (TI “SARS-CoV-2” OR AB “SARS-CoV-2” OR MH “COVID-19)) **n=1** | (((TOPIC “diabetes mellitus” AND “pregnancy) OR (TOPIC “gestational diabetes mellitus”)) AND ((TOPIC “COVID-19” OR TOPIC “SARS-CoV-2”))) **n=10** |  |

*Supplementary Table 2. Included Articles.*

|  | **Reference** |
| --- | --- |
|  | Abbas AM, Ahmed OA, Shaltout AS. COVID-19 and maternal pre-eclampsia: A synopsis. Scand J Immunol 2020;92:e12918. |
|  | Ahn DT. The COVID-19 Pandemic: A "Tech"-tonic Shift Toward Virtual Diabetes Care. J Diabetes Sci Technol 2020;14:708–9. |
|  | Allotey J, Stallings E, Bonet M, et al. Clinical manifestations, risk factors, and maternal and perinatal outcomes of coronavirus disease 2019 in pregnancy: living systematic review and meta-analysis. BMJ 2020;370:m3320. |
|  | Al-Salameh A, Lanoix J-P, Bennis Y, et al. Characteristics and outcomes of COVID-19 in hospitalized patients with and without diabetes. Diabetes/metabolism research and reviews 2020. |
|  | Alzamora MC, Paredes T, Caceres D, Webb CM, Valdez LM, La Rosa M. Severe COVID-19 during Pregnancy and Possible Vertical Transmission. American Journal of Perinatology 2020;37:861–5. (https://www.embase.com/search/results?subaction=viewrecord&id=L632151744&from=export). |
|  | Azar WS, Njeim R, Fares AH, et al. COVID-19 and diabetes mellitus: how one pandemic worsens the other. Reviews in Endocrine & Metabolic Disorders 2020. |
|  | Aziz A, Zork N, Aubey JJ, et al. Telehealth for High-Risk Pregnancies in the Setting of the COVID-19 Pandemic. American Journal of Perinatology 2020;37:800–8. (https://www.embase.com/search/results?subaction=viewrecord&id=L632151753&from=export). |
|  | Bahadur G, Homburg R, Yoong W, et al. Adverse outcomes in SAR-CoV-2 (COVID-19) and SARS virus related pregnancies with probable vertical transmission. JBRA Assist Reprod 2020;24:351–7. |
|  | Bajgain KT, Badal S, Bajgain BB, Santana MJ. Prevalence of comorbidities among individuals with COVID-19: A rapid review of current literature. American Journal of Infection Control 2020. |
|  | Braems G. Fetal hypoxemia on a molecular level: adaptive changes in the hypothalamic–pituitary–adrenal (HPA) axis and the lungs. European Journal of Obstetrics & Gynecology and Reproductive Biology 2003;110:S63-S69. |
|  | Cavezzi A, Troiani E, Corrao S. COVID-19: hemoglobin, iron, and hypoxia beyond inflammation. A narrative review. Clin Pract 2020;10:1271. |
|  | Chen Y, Yang D, Cheng B, et al. Clinical Characteristics and Outcomes of Patients With Diabetes and COVID-19 in Association With Glucose-Lowering Medication. Diabetes Care 2020;43:1399–407. |
|  | Farooqi MH. Tele-Management of Diabetes in the Post-COVID-19 Era. Dubai Diabetes Endocrinol J 2020:1–3. |
|  | Ferraiolo A, Barra F, Kratochwila C, et al. Report of Positive Placental Swabs for SARS-CoV-2 in an Asymptomatic Pregnant Woman with COVID-19. Medicina (Kaunas) 2020;56. |
|  | Filardi T, Morano S. COVID-19: is there a link between the course of infection and pharmacological agents in diabetes? J Endocrinol Invest 2020;43:1053–60. |
|  | Gentile S, Strollo F, Mambro A, Ceriello A. COVID-19, ketoacidosis, and new-onset Diabetes: might we envisage any cause-effect relationships among them? Diabetes Obes Metab 2020. |
|  | German Diabetes Association. „Aus der Krise lernen: Digitalisierung und Telemedizin nutzen und Versorgung von Patienten mit Diabetes mellitus verbessern – auch nach der Corona-Pandemie, 2020. (https://www.deutsche-diabetes-gesellschaft.de/pressekonferenzen/endstation-notstand-wie-wir-die-weichen-fuer-eine-gute-diabetesversorgung-von-morgen-stellen-1). |
|  | Gerste RD. Thrombose-Pathogenese: Endotheldysfunktion bei COVID-19. Dtsch Arztebl 2020;117:22–3. |
|  | Gracia-Ramos AE. Is the ACE2 Overexpression a Risk Factor for COVID-19 Infection? Archives of Medical Research 2020;51:345–6. (https://www.embase.com/search/results?subaction=viewrecord&id=L2005525572&from=export). |
|  | Guo W, Li M, Dong Y, et al. Diabetes is a risk factor for the progression and prognosis of COVID-19. Diabetes/metabolism research and reviews 2020:e3319. |
|  | Hartmann-Boyce J, Morris E, Goyder C, et al. Diabetes and COVID-19: Risks, Management, and Learnings From Other National Disasters. Diabetes Care 2020;43:1695–703. |
|  | Hecht JL, Quade B, Deshpande V, et al. SARS-CoV-2 can infect the placenta and is not associated with specific placental histopathology: a series of 19 placentas from COVID-19-positive mothers. Mod Pathol 2020. |
|  | Iqbal A, Prince LR, Novodvorsky P, et al. Effect of Hypoglycemia on Inflammatory Responses and the Response to Low-Dose Endotoxemia in Humans. J Clin Endocrinol Metab 2019;104:1187–99. |
|  | Katulanda P, Dissanayake HA, Ranathunga I, et al. Prevention and management of COVID-19 among patients with diabetes: an appraisal of the literature. Diabetologia 2020;63:1440–52. |
|  | Kleinwechter H, Laubner K. Coronavirus disease 2019 (COVID-19) and pregnancy: Overview and report of the first German case with COVID-19 and gestational diabetes. Diabetologe 2020;16:242–6. (https://www.embase.com/search/results?subaction=viewrecord&id=L2004630640&from=export). |
|  | Kleinwechter H. Diabetes and pregnancy—update 2020. Diabetologe 2020;16:470–7. (https://www.embase.com/search/results?subaction=viewrecord&id=L2005018281&from=export). |
|  | Li B, Yang J, Zhao F, et al. Prevalence and impact of cardiovascular metabolic diseases on COVID-19 in China. Clinical Research in Cardiology 2020;109:531–8. (https://www.embase.com/search/results?subaction=viewrecord&id=L2004443220&from=export). |
|  | Liu H, Wang L-L, Zhao S-J, Kwak-Kim J, Mor G, Liao A-H. Why are pregnant women susceptible to COVID-19? An immunological viewpoint. J Reprod Immunol 2020;139:103122. |
|  | Liu Y, Chen H, Tang K, Guo Y. Clinical manifestations and outcome of SARS-CoV-2 infection during pregnancy. The Journal of infection 2020. |
|  | McIntyre HD, Moses RG. The Diagnosis and Management of Gestational Diabetes Mellitus in the Context of the COVID-19 Pandemic. Diabetes Care 2020;43:1433–4. (https://www.embase.com/search/results?subaction=viewrecord&id=L2006913026&from=export). |
|  | Moradi F, Ghadiri-Anari A, Enjezab B. COVID-19 and self-care strategies for women with gestational diabetes mellitus. Diabetes Metab Syndr 2020;14:1535–9. (https://www.embase.com/search/results?subaction=viewrecord&id=L2007473075&from=export). |
|  | Narang K, Enninga EAL, Gunaratne MDSK, et al. SARS-CoV-2 Infection and COVID-19 During Pregnancy: A Multidisciplinary Review. Mayo Clinic Proceedings 2020;95:1750–65. (http://www.sciencedirect.com/science/article/pii/S0025619620305164). |
|  | Nouhjah S, Jahanfar S, Shahbazian H. Temporary changes in clinical guidelines of gestational diabetes screening and management during COVID-19 outbreak: A narrative review. Diabetes Metab Syndr 2020;14:939–42. (https://www.embase.com/search/results?subaction=viewrecord&id=L2006842835&from=export). |
|  | Pal R, Bhadada SK. COVID-19 and diabetes mellitus: An unholy interaction of two pandemics. Diabetes Metab Syndr 2020;14:513–7. |
|  | Pal R, Bhansali A. COVID-19, diabetes mellitus and ACE2: The conundrum. Diabetes Res Clin |
|  | Penfield CA, Brubaker SG, Limaye MA, et al. Detection of severe acute respiratory syndrome coronavirus 2 in placental and fetal membrane samples. American Journal of Obstetrics and Gynecology MFM 2020;2:100133. |
|  | Rubino F, Amiel SA, Zimmet P, et al. New-Onset Diabetes in Covid-19. N Engl J Med 2020;383:789–90. (https://www.embase.com/search/results?subaction=viewrecord&id=L632065623&from=export). |
|  | Schwartz DA. An Analysis of 38 Pregnant Women with COVID-19, Their Newborn Infants, and Maternal-Fetal Transmission of SARS-CoV-2: Maternal Coronavirus Infections and Pregnancy Outcomes. Arch Pathol Lab Med 2020. |
|  | Simon M, Sarkar N, Kumaran S, Chittake A, Purandare V, Unnikrishnan A. Telemedicine for the initial management of newly diagnosed gestational diabetes in the pandemic period: A report of three case studies. J Diabetol 2020;11:144. |
|  | Stegenga ME, van der Crabben SN, Blümer RME, et al. Hyperglycemia enhances coagulation and reduces neutrophil degranulation, whereas hyperinsulinemia inhibits fibrinolysis during human endotoxemia. Blood 2008;112:82–9. |
|  | Tsai S, Clemente-Casares X, Zhou AC, et al. Insulin Receptor-Mediated Stimulation Boosts T Cell Immunity during Inflammation and Infection. Cell Metab 2018;28:922-934.e4. |
|  | Verity R, Okell LC, Dorigatti I, et al. Estimates of the severity of coronavirus disease 2019: a model-based analysis. The Lancet Infectious Diseases 2020;20:669–77. |
|  | Vivanti AJ, Vauloup-Fellous C, Prevot S, et al. Transplacental transmission of SARS-CoV-2 infection. Nat Commun 2020;11:3572. |
|  | Wei Z-Y, Geng Y-J, Huang J, Qian H-Y. Pathogenesis and management of myocardial injury in coronavirus disease 2019. European Journal of Heart Failure 2020. |
|  | Xie J, Covassin N, Fan Z, et al. Association Between Hypoxemia and Mortality in Patients With COVID-19. Mayo Clinic Proceedings 2020;95:1138–47. |
|  | Yang H, Wang C, Poon LC. Novel coronavirus infection and pregnancy. Ultrasound Obstet Gynecol 2020;55:435–7. |
|  | Yang J, Zheng Y, Gou X, et al. Prevalence of comorbidities and its effects in coronavirus disease 2019 patients: A systematic review and meta-analysis. Int J Infect Dis 2020;94:91–5. (https://www.embase.com/search/results?subaction=viewrecord&id=L2005625485&from=export). |
|  | Yang J-K, Lin S-S, Ji X-J, Guo L-M. Binding of SARS coronavirus to its receptor damages islets and causes acute diabetes. Acta Diabetol 2010;47:193–9. |
|  | Zaigham M, Andersson O. Maternal and perinatal outcomes with COVID-19: A systematic review of 108 pregnancies. Acta Obstet Gynecol Scand 2020;99:823–9. (https://www.embase.com/search/results?subaction=viewrecord&id=L2004697896&from=export). |

*Supplementary Figure 1. PRISMA flow chart: search and selection process (modified according to Moher et al. 2009).*

**COVID-19 and Diabetic Pregnancies (III)**
Records identified in September 2020 (n=70)

PubMed: n=10
CINAHL: n=1
Cochrane Library: n=0
Web of Science Core Collection: n=10
EMBASE: n=49

**COVID-19 and Pregnancy (II)**
Records identified in September 2020 (n=1,516)

PubMed: n=523
CINAHL: n=149
Cochrane Library: n=0
Web of Science Core Collection: n=381
EMBASE: n=463

**COVID-19 and Diabetes
Mellitus (I)**
Records identified in September 2020 (n=2,382)

PubMed: n=396
CINAHL: n=63
Cochrane Library: n=8
Web of Science Core Collection: n=195
EMBASE: n=1720

Records after duplicates removed
n=1,938 (I), n=985 (I), n=70 (III)

Selection of suitable articles on the following subtopics:

- Pathophysiology
- “Perinatal/trans-generational” programming
- Screening
- Management
- Clinical outcomes and features

Articles excluded:
(n=2958)

- Genereally wrong topic
- Not Diabetes in Pregnancy
- Not COVID-19

Manual research via Google Scholar and reference lists
(n=10)

Records screened
(n=3,003)

Articles incuded in synthesis
(n=49)
